# Comparing the acute responses between a manual and automated blood flow restriction system

**DOI:** 10.3389/fphys.2024.1409702

**Published:** 2024-06-14

**Authors:** Daphney M. Carter, Matthew A. Chatlaong, William M. Miller, J. Barnes Benton, Matthew B. Jessee

**Affiliations:** ^1^ Wellstar College of Health and Human Services, Department of Exercise Science and Sport Management, Kennesaw State University, Kennesaw, GA, United States; ^2^ Applied Human Health and Physical Function Laboratory, School of Applied Science, Department of Health, Exercise Science, and Recreation Management, The University of Mississippi, University, MS, United States; ^3^ College of Education and Health Sciences, School of Health Sciences, University of Evansville, Evansville, IN, United States; ^4^ School of Medicine, University of Mississippi Medical Center, Jackson, MS, United States

**Keywords:** muscle thickness, ultrasound, hemodynamic, resistance, perception, strength, vascular, kaatsu

## Abstract

The purpose of this study was to compare acute responses between manual and automated blood flow restriction (BFR) systems.

**Methods:**

A total of 33 individuals completed this study. On visit 1, arterial occlusion pressure (AOP, mm Hg), cardiovascular responses, and discomfort (RPE-D) were measured with each BFR system at rest. On visit 2, unilateral bicep curls were completed [30% one-repetition maximum; 50% AOP] with one system per arm. Muscle thickness (MT, cm) and maximal force (N) were assessed before (pre), immediately (post-0), 5 min (post-5), and 10 min (post-10) post-exercise. Ratings of perceived exertion (RPE-E) and ratings of perceived discomfort (RPE-D) were assessed throughout the exercise. AOP and repetitions were compared with Bayesian paired t-tests. Other outcomes were compared with Bayesian RMANOVAs. BF_10_ represents the likelihood of the best model vs. the null. The results are presented as mean ± SD.

**Results:**

Supine cardiovascular responses and RPE-D were similar for manual and automated (all BF_10_ ≤ 0.2). Supine AOP for manual (157 ± 20) was higher than that of automated (142 ± 17; BF_10_ = 44496.0), but similar while standing (manual: 141 ± 17; automated: 141 ± 22; BF_10_ = 0.2). MT (time, BF_10_ = 6.047e + 40) increased from Pre (3.9 ± 0.7) to Post-0 (4.4 ± 0.8; BF_10_ = 2.969e + 28), with Post-0 higher than Post-5 (4.3 ± 0.8) and Post-10 (4.3 ± 0.8; both BF_10_ ≥ 275.2). Force (time, BF_10_ = 1.246e + 29) decreased from Pre (234.5 ± 79.2) to Post-0 (149.8 ± 52.3; BF_10_ = 2.720e + 22) and increased from Post-0 to Post-5 (193.3 ± 72.7; BF_10_ = 1.744e + 13), with Post-5 to Post-10 (194.0 ± 70.6; BF_10_ = 0.2) being similar. RPE-E increased over sets. RPE-D was lower for manual than automated. Repetitions per set were higher for manual (Set 1: 37 ± 18; Set 4: 9 ± 5) than automated (Set 1: 30 ± 7; Set 4: 7 ± 3; all BF_10_ ≥ 9.7).

**Conclusion:**

Under the same relative pressure, responses are mostly similar between BFR systems, although a manual system led to lower exercise discomfort and more repetitions.

## 1 Introduction

With the increasing popularity of blood flow restriction (BFR) exercise, many systems have become available, ranging from simple wraps to automated pneumatic systems. The goal of BFR is to temporarily reduce arterial blood flow to the exercising muscles, thereby inducing fatigue sooner than the same exercise without BFR (Kolind et al., 2022). Muscular fatigue can be measured by the decline in muscular force following exercise (Nocella et al., 2011). Exercising with these low loads when compared to traditional loads is advantageous for clinical populations due to a lesser joint strain. When training with BFR, the increase in BFR pressure typically decreases the workload necessary for increases in muscle size and strength (Jessee et al., 2018c). A proposed mechanism for muscular adaptations is muscle swelling (Jessee et al., 2018a), which can be measured by acute changes in muscle thickness (Loenneke et al., 2012b).

Current recommendations for BFR involve applying a pressure based on individual arterial occlusion pressure (AOP) (Patterson et al., 2019). This is typically the pressure at which arterial and venous blood flow are occluded and is largely influenced by individual characteristics such as limb circumference (Loenneke et al., 2012a; Jessee et al., 2016) and system characteristics such as cuff width (Loenneke et al., 2012a; Loenneke et al., 2013b). By applying a recommended pressure ranging from 40% to 80% AOP, it is assumed that there would be arterial blood flow toward the exercising muscles while completing sets to failure or the 30 × 15 × 15 × 15 repetition protocol.

A manual system (Hokanson AG101, E20 Cuff Inflator, and an MD6 Doppler probe) is commonly used in research to apply BFR based on the AOP. To assess AOP using a manual system, the pressure of the cuff needs to be increased until a detectable pulse (usually via the Doppler probe) distal to the cuff is no longer present (Jessee et al., 2018a; Abe et al., 2019). Previous research has shown that use of relative pressures based on a percentage of AOP and/or exercising until momentary failure seems to negate differences in the exercise response between different cuff widths (Bell et al., 2020) and manual systems (Buckner et al., 2017). In contrast to manual systems, automated systems (e.g., Delfi Personalized Tourniquet System) have the capability to estimate the AOP and apply BFR without additional equipment (Hughes et al., 2018; McEwen et al., 2019), making them desirable and common in clinical settings.

Since the recommended application of BFR is reliant on AOP, if the AOP measurement is over or underestimated between systems this could alter the stimulus and the associated response, potentially limiting the translation of evidence from the lab to clinical settings. It is unclear whether BFR applied with a manual or automated system would elicit different responses during rest, exercise, or recovery. Thus, our primary purpose was to compare the responses between a manual system (commonly used in research settings) and an automated system (commonly used in clinical settings) when using the same BFR protocol. If these systems produce similar outcomes, then researchers may integrate the knowledge from both systems to further improve the application of BFR exercise in both clinical and research settings. Based on previous literature utilizing system-specific AOP, our hypothesis was that when using an individualized BFR pressure, the resting and exercise responses would be similar between systems.

## 2 Materials and methods

### 2.1 Ethics approval

Participants eligible for the study provided informed consent prior to data collection. This study conformed to the standards set by the Declaration of Helsinki and was approved by the University of Mississippi’s institutional review board (#21-040).

### 2.2 Participants

A total of 34 participants participated in the study. Participants were excluded from the study if they were less than 18 years of age, took medication that would influence heart rate or blood pressure, used nicotine regularly within the last 6 months, or not upper body resistance trained. Participants were also ineligible if they were at an increased risk of thromboembolism by having more than one of the following seven risk factors (adapted from Motykie et al., 2000): taking hormone birth control (excluding progestin-only); diagnosed Crohn’s or inflammatory bowel disease; past fracture of a hip, pelvis, or femur; major surgery within the last 6 months; diagnosed varicose veins; personal/family history of deep vein thrombosis; or personal/family history of pulmonary embolism. Participants were asked to avoid caffeine and food for 2 h prior to each visit. This timeframe was chosen because blood flow velocity, systolic blood pressure, diastolic blood pressure, heart rate, and post-occlusive reactive hyperemia are similar in habitual caffeine users when abstaining or consuming caffeine (Chatlaong et al., 2024). Participants were also asked to avoid alcohol for 24 h and upper body exercise for 48 h prior to each visit. Additionally, participants were asked to maintain their current nutrition and sleep habits during the course of the study.

### 2.3 Protocol overview

**FIGURE 1:**
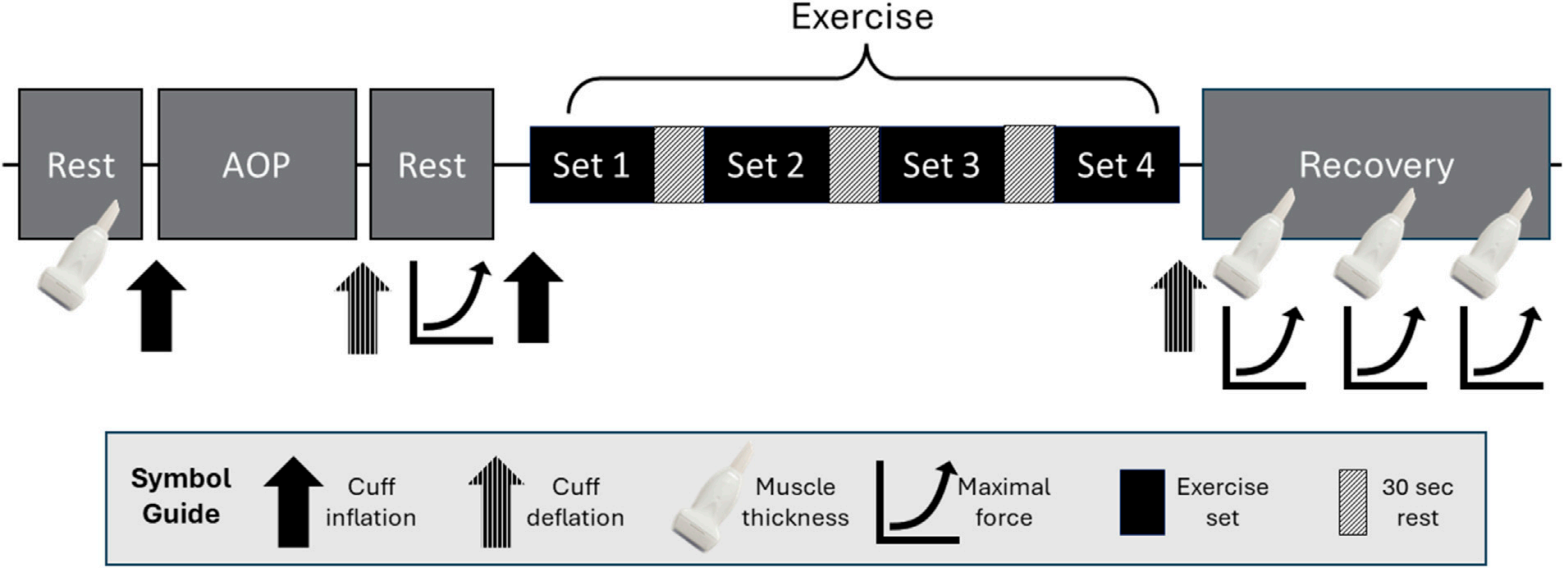
Protocol order for Visit 2 starting with the initial resting measurements of muscle thickness in both arms. Following this, measures were specific to the first arm and system used. Standing arterial occlusion pressure (AOP) was measured, followed by ratings of perceived exertion (RPE-E) and discomfort (RPE-D), which are not shown in this figure. Immediately after, there were two attempts for maximum voluntary isometric contraction (MVIC). Blood flow restriction exercise started immediately after cuff inflation with RPE-E, RPE-D, and repetitions recorded for each set. Following the exercise protocol, muscle thickness and MVIC were measured immediately and then again after 5 and 10 min. These procedures were repeated in the other arm with the second system starting with the initial rest period.

Using a within-participant design, participants visited the laboratory twice (separated by 2–14 days). On visit 1, all resting supine outcomes were measured with each system and compared. On visit 2, all exercise outcomes were measured with a system attached per arm and compared. During visit 1, informed consent was obtained, and then height and body mass were measured. A 5-min rest period was selected based on previous recommendations (Whelton et al., 2018). Then, participants had a 5-min supine rest in a dark room with the cuff of the first system on their right arm (abducted approximately 90° from the body, resting on a table). The AOP was measured, followed by another 5-min rest. Blood flow, ratings of perceived discomfort (RPE-D), heart rate, and percent oxygen saturation were measured and then repeated after 1 min. The two measures were averaged for obtaining baseline values. The cuff was then inflated to 50% of the AOP for 1 min, and all measures were taken a third time. Starting with the initial 5-min rest, this process was repeated with the cuff of the second system on the same arm. Change scores for each variable were calculated as the difference between 50% AOP and baseline. Visit 1 was then concluded with one-repetition maximum testing (1RM). For visit 2 (Figure 1), participants were seated for 5 min, and then pre-exercise (Pre) upper arm muscle thickness was measured in both arms. The AOP was assessed with the first system and arm, while the opposite arm was allowed to relax at the participant’s side. Then, participants had another 5-min seated rest. While standing, Pre measures of RPE-D and ratings of perceived exertion (RPE-E) were assessed, followed by maximum voluntary isometric contraction (MVIC). Then, the first exercise protocol was begun. Repetitions, RPE-D, and RPE-E were recorded throughout. Immediately following exercise (Post-0), the cuff was deflated, the MVIC was conducted, the cuff was removed, and muscle thickness was assessed. At 5 min (Post-5) and 10 min (Post-10) post-exercise, muscle thickness was assessed, followed by an MVIC. The same protocol was repeated in the other arm with the second system, starting with the initial 5-min seated rest. The order of systems used was randomized and counterbalanced for both visits.

### 2.4 Arterial occlusion pressure

The AOP was measured with a BFR cuff applied to the uppermost portion of the arm. For the manual system, the cuff (SC5, Hokanson, Bellevue, WA) was inflated (EC20 Rapid Cuff Inflator, Hokanson, Bellevue, WA) to a pressure of 50 mm Hg initially, and the pressure was increased at a steady pace until cessation of an audible pulse (MD6, Hokanson, Bellevue, WA) at the wrist, and then the cuff was immediately deflated. This method of measuring the AOP is considered reliable (Karanasios et al., 2022) and valid when compared to the ultrasound Doppler AOP (Laurentino et al., 2020). For the automated system, the AOP was automatically assessed by the system (PTS, Delfi, Vancouver, BC). If the system reported a noisy signal, the cuff (Easi-Fit BFR 18 in., Delfi, Vancouver, BC) was repositioned, and AOP was reassessed. Since AOP differs depending on body position, it was measured in the position in which BFR would be applied (Sieljacks et al., 2018). On visit 1, AOP was assessed in the supine position, and on visit 2 while standing prior to exercise. The BFR pressure of 50% AOP was selected based on current BFR exercise recommendations (Patterson et al., 2019).

### 2.5 Blood flow

Blood flow measurements were conducted as in previous studies (Mouser et al., 2017; Stanford et al., 2022). A linear array ultrasound probe (LA3-14AD, Samsung, Danvers, MA), coated in transmission gel, was placed proximal to the antecubital crest on the right arm. The probe and ultrasound settings were adjusted until the brachial artery spanned longitudinally across the screen. Following the protocol, blood flow velocity was determined using a limited trace of five cardiac cycles from screen capture. If all five cardiac cycles were not viable, the next available viable cardiac cycles were used. When no cardiac cycles were viable, the data were excluded from analysis. Ultrasound (HS60, Samsung, Danvers, MA) digital calipers were used to determine the arterial diameter. Following the measurement of the limited trace and arterial diameter, blood flow volume was computed using the manufacturer-provided ultrasound imaging software.

### 2.6 Oxygen saturation and heart rate

A pulse oximeter (Pro Series 500DL, Zacurate, Stafford, TX) was placed on the right index finger prior to the first 5-min supine rest. Oxygen saturation and heart rate were recorded for each resting measurement.

### 2.7 One-repetition maximum test

Maximal dynamic strength of the elbow flexors was evaluated on each arm using a 1RM test with a free-weight dumbbell, while the participant was positioned with their heels and back against a wall. 1RM was defined as the maximal weight lifted (biceps curl) one time with proper form. The trial began with a warm-up of 5–10 repetitions at < 50% of an estimated 1RM. After a brief rest period, the load was increased to ∼75% of the estimated maximum for one repetition. After another brief rest period, a small increase in weight was added to the load, and a 1RM was attempted. An attempt was considered successful if the weight was lifted through a full range of motion with proper form. Following successful attempts, the weight was increased. If an attempt was unsuccessful, the weight was decreased. Attempts alternated between arms, with 90 s of standing rest separating each attempt. Thus, the same arm would have 3-min rest before another attempt. During each 1RM trial, researchers provided verbal encouragement, and the highest achieved 1RM was recorded for each arm. 1RM was typically determined within five attempts, except in two instances where there were six attempts. There is good-to-excellent reliability of the 1RM test across protocols that vary from 3 to 8 attempts and 1–5 min of rest between attempts (Grgic et al., 2020).

### 2.8 Muscle thickness

While standing, the anterior muscle thickness of the upper arm was assessed at 70% of the distance from the acromion process of the scapula to the lateral epicondyle of the humerus. This distance was measured using an inelastic tape measure and marked with a semi-permanent marker, and then a horizontal line was drawn across the biceps for the anterior measure. A linear array ultrasound probe coated in transmission gel was lightly placed against the skin perpendicular to the midline of the muscle. Then, two images were taken, and the distance from the top of the bone to the muscle-adipose interface (i.e., muscle thickness) was measured with the ultrasound on-screen calipers. If the difference between the two measures was greater than 0.2 cm, a third image was taken. The closest two values were averaged together and used for analysis.

### 2.9 Rating of perceived discomfort and exertion

Separate 11-point scales were used to assess RPE-D and RPE-E. Standardized scripts were read and shown to each participant (Steele et al., 2016). RPE-D was assessed on visit 1 during supine measurements and on visit 2 at Pre and 20 s after each set of exercise. RPE-E was assessed on visit 2 at Pre and immediately following each set. After the last set of exercise, both RPE-D and RPE-E were assessed immediately.

### 2.10 Maximal voluntary isometric contraction force test

The maximal isometric strength of the elbow flexors was evaluated using a custom-made apparatus. Participants were instructed to maintain a standing upright posture with their arm by their side at approximately 90° of elbow flexion. They were given a handle that was connected to a tensiometer (SM-500, Interface, Scottsdale, AZ; Delsys, Natick, MA), with the other end of the tensiometer connected to a rigid constraint. Participants were instructed to grab the handle and pull against the tensiometer as hard as possible for 3–5 s with ongoing verbal encouragement. Participants completed two trials, with 1 min of rest between each trial. The MVIC was considered the peak value achieved during the trials, with any artifacts excluded (LabVIEW, National Instruments, Austin, TX). The difference between the offset (1 s mean value prior to contraction) and the MVIC was converted to force and used for data analysis.

### 2.11 Resistance exercise protocol

Four sets of unilateral bicep curls were performed to momentary failure at 30% 1RM with BFR. The exercise was performed in the same position as the 1RM test. Prior to exercise, the BFR cuff was fitted to the upper portion of the exercising arm and inflated to 50% AOP until all sets were completed. Participants were instructed to lift and lower the weight in a controlled manner to a metronome at 1.5 s for the concentric phase and 1.5 s for the eccentric phase. For each set, the participant was asked to complete as many repetitions as possible, while strong verbal encouragement was provided. Sets were terminated when the participant stopped, could no longer control the weight in sync with the metronome (>2 repetitions), or could no longer complete a full range of motion. Sets were interspersed by a 30 s rest, while the cuff remained inflated.

### 2.12 Statistical analysis

To test for differences in the AOP (separate comparisons for supine and standing) and change in scores for RPE-D, oxygen saturation, and blood flow, Bayesian-paired sample T-tests (JASP, 0.17.3, Amsterdam, NL) were used. To compare muscle thickness, force, RPE-D, and RPE-E between systems across time points, Bayesian repeated-measures ANOVAs with factors of condition and time were used. Repetitions were compared with Bayesian-paired sample T-tests between systems for each set. Initially, the most probable model was determined. Then, the most probable model was compared to the null model based on the Bayes factors (BF_10_). If the null model was not the most probable model, then follow-up comparisons were conducted. Levels of evidence associated with the BF_10_ are reported, with values less than 1 favoring the null hypothesis and greater than 1 favoring the alternative hypothesis (Wagenmakers et al., 2018). An uninformed prior was used for all analyses, and BF_10_ are provided with their respective error %. Our resultant sample size is similar to that in other studies (Rossow et al., 2012; Jessee et al., 2017a; Mouser et al., 2017; Bordessa et al., 2021), and data collection was stopped when a BF_10_ of more than moderate evidence (<.333 or >3) was reached. Data are reported as mean ± SD in the results.

## 3 Results

### 3.1 Participants

A total of 33 participants completed the study (female = 14; right-handed = 26; age = 26 ± 7 years; height = 170.6 ± 7.9 cm; and weight = 71.5 ± 14.7 kg). However, some data points were excluded from the final analyses due to missing data, poor signal, equipment malfunction, etc. The sample size for each measure is provided. One participant developed bruising on their arm following the second exercise bout, which appeared similar to a previous case report (Kondo, 2018).

### 3.2 Resting cardiovascular response and discomfort

In the supine position, manual AOP was higher when compared to that in the automated system (BF_10_ = 44496.028; error = 3.631e − 10%). The changes in blood flow (BF_10_ = 0.208; error = 0.036%), oxygen saturation (BF_10_ = 0.202; error = 0.039%), heart rate (BF_10_ = 0.200; error = 0.039%), and RPE-D (BF_10_ = 0.198; error = 0.039%) were similar between systems. All the data are provided in Table 1.

**TABLE 1:** Supine measures of cardiovascular response and RPE-D.

	AOP	Δ Blood flow	Δ Oxygen saturation	Δ Heart rate	Δ RPE-D
Manual	157 ± 20	−28.41 ± 27.10	−1 ± 1	0 ± 4	1 ± 1
Automated	142 ± 17*	−26.72 ± 29.09	−1 ± 1	0 ± 6	1 ± 1

Visit 1 measures of arterial occlusion pressure (AOP) (mm Hg), Δ blood flow (mL/min), Δ oxygen saturation (%), Δ heart rate (bpm), and Δ ratings of perceived discomfort (RPE-D) (A.U.); RPE-D are presented as mean ± SD. Δ is the change from the averaged baseline value to the value 1 min following cuff inflation to 50% AOP, and * indicates a difference between systems (BF_10_ > 3). For all variables, *n* = 33, except Δ blood flow, for which *n* = 31.

### 3.3 Arterial occlusion pressure (mm Hg; *n* = 32)

There was evidence that standing AOP was similar between the manual system (141 ± 17) and automated system (141 ± 22; BF_10_ = 0.190; error = 0.038%).

### 3.4 Muscle thickness (cm; *n* = 32)

**FIGURE 2:**
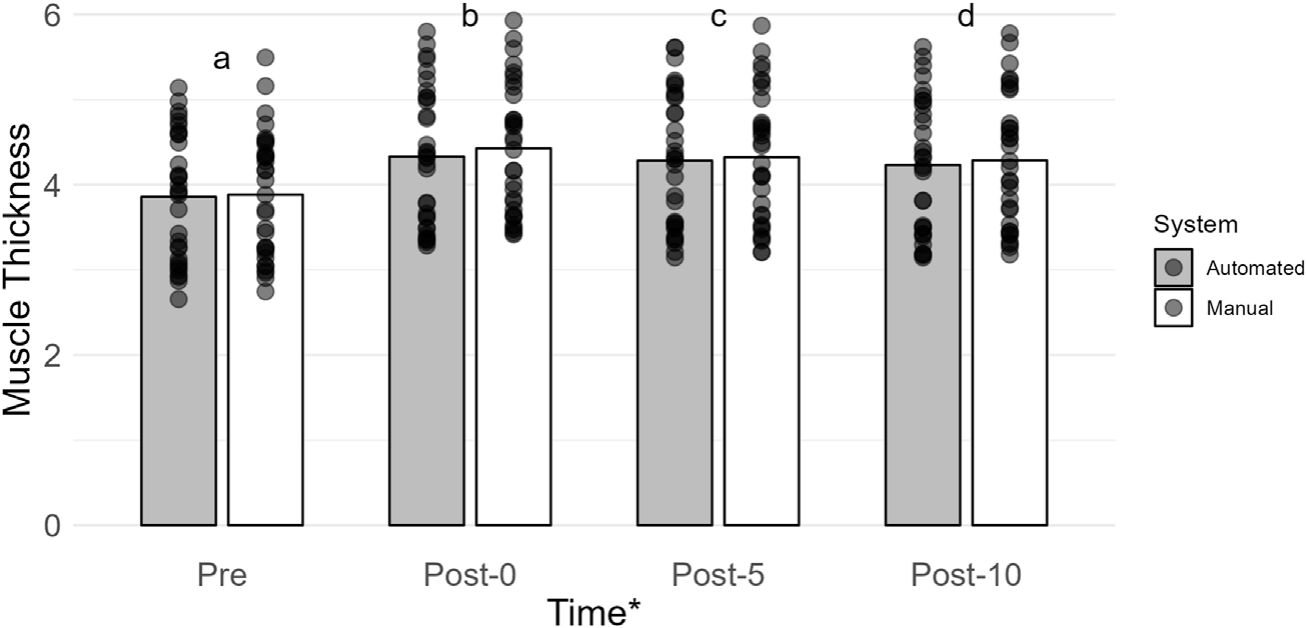
Mean muscle thickness (cm) is shown by vertical bars (automated, gray; manual, white), and individual scores are shown with semi-transparent circles. A * indicates a main effect of time. If a time point has a different lowercase letter, then there is more than moderate evidence (BF_10_ > 3), indicating a difference across time. Pre, before exercise; Post-0, immediately after exercise, Post-5, 5 min after exercise; Post-10, 10 min after exercise.

The main effect of time was the most probable model (BF_10_ = 6.047e + 40; error = 2.494%; Figure 2). Collapsed across conditions, muscle thickness was lower at Pre (3.87 ± 0.73) when compared to Post-0 (4.38 ± 0.78; BF_10_ = 2.969e + 28), Post-5 (4.30 ± 0.77; BF_10_ = 1.673e + 28), and Post-10 (4.26 ± 0.77; BF_10_ = 3.300e + 25). Muscle thickness was higher at Post-0 than at Post-5 (BF_10_ = 26721.067) and Post-10 (BF_10_ = 2.239e + 9). Muscle thickness at Post-5 was higher than that at Post-10 (BF_10_ = 275.200). All *post hoc* comparison errors were ≤ 7.872e − 7%.

### 3.5 Ratings of perceived exertion (A.U.; *n* = 31)

**FIGURE 3:**
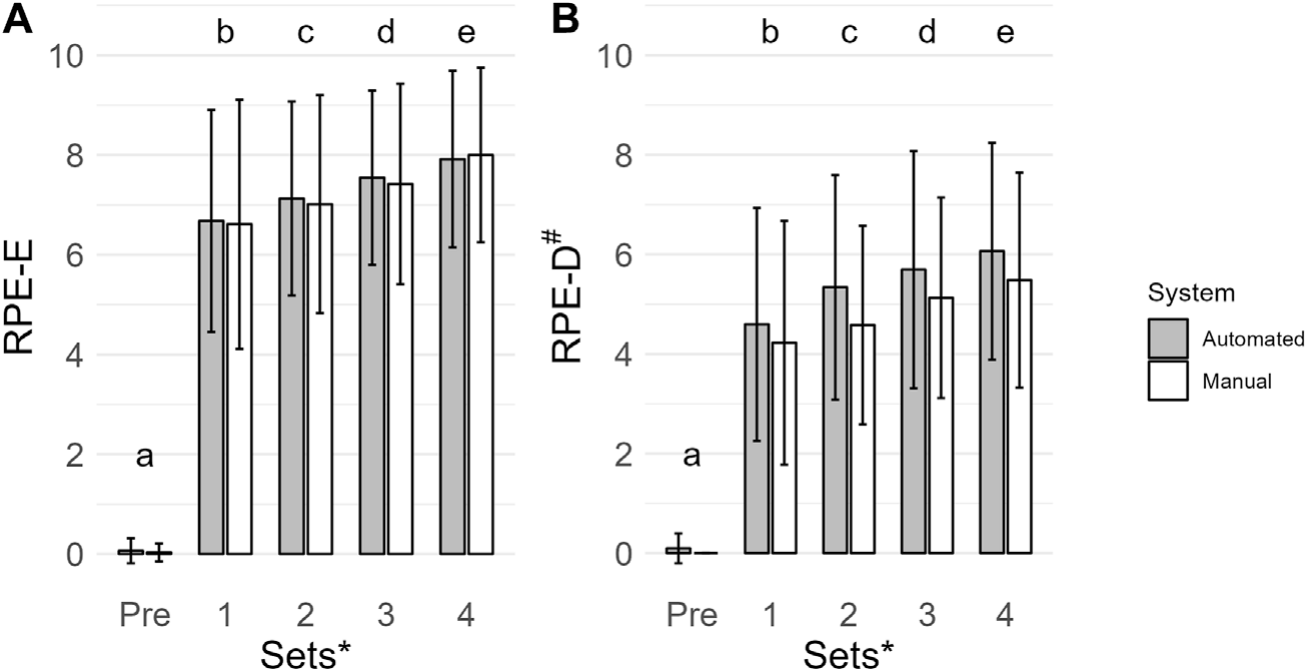
Mean ratings of perceived exertion (RPE-E, A. U.) **(A)** and ratings of perceived discomfort (RPE-D, A. U.) **(B)** are shown by vertical bars (automated, gray; manual, white), and error bars show the standard deviation. A # indicates a main effect of condition for RPE-D with a lower value for the manual system. A * indicates a main effect of time for RPE-E and RPE-D. If a time point has a different lowercase letter, then there is more than moderate evidence (BF_10_ > 3), indicating a difference across time. Pre, before exercise.

The main effect of time was the most probable model (BF_10_ = 4.012e + 60; error = 1.430%; Figure 3A). Collapsed across conditions, Pre (0 ± 0) had a lower RPE-E than set 1 (7 ± 2; BF_10_ = 2.498e + 27), set 2 (7.±2; BF_10_ = 7.514e + 31), set 3 (7 ± 2; BF_10_ = 5.476e + 35), and set 4 (8 ± 2; BF_10_ = 1.051e + 39). Set 1 was lower than set 2 (BF_10_ = 6.197), set 3 (BF_10_ = 18515.416), and set 4 (BF_10_ = 1.618e + 7). Set 2 was lower than set 3 (BF_10_ = 485.741) and set 4 (BF_10_ = 9.741e + 6). Set 3 was lower than set 4 (BF_10_ = 1691.749). All *post hoc* comparison errors were ≤ 1.153e − 6%.

### 3.6 Ratings of perceived discomfort (A.U.; *n* = 31)

The main effect of time and condition was the most probable model (BF_10_ = 1.665e + 42; error = 1.710%; Figure 3B). Collapsed across conditions, Pre (0 ± 0) had a lower RPE-D than set 1 (4 ± 2; BF_10_ = 2.206e + 18), set 2 (5 ± 2; BF_10_ = 9.553e + 22), set 3 (5 ± 2; BF_10_ = 1.982e + 24), and set 4 (6 ± 2; BF_10_ = 1.700e + 26). Set 1 was lower than set 2 (BF_10_ = 91.579), set 3 (BF_10_ = 258415.461) and set 4 (BF_10_ = 1.108e + 6). Set 2 was lower than set 3 (BF_10_ = 190.313) and set 4 (BF_10_ = 53067.977). Set 3 was lower than set 4 (BF_10_ = 5.830). Collapsed across time, RPE-D were lower when exercising with the manual system (4 ± 2) compared to the automated system (4 ± 2; BF_10_ = 525.886). All *post hoc* comparison errors were ≤ 1.954e − 7%.

### 3.7 Force (N; *n* = 31)

**FIGURE 4:**
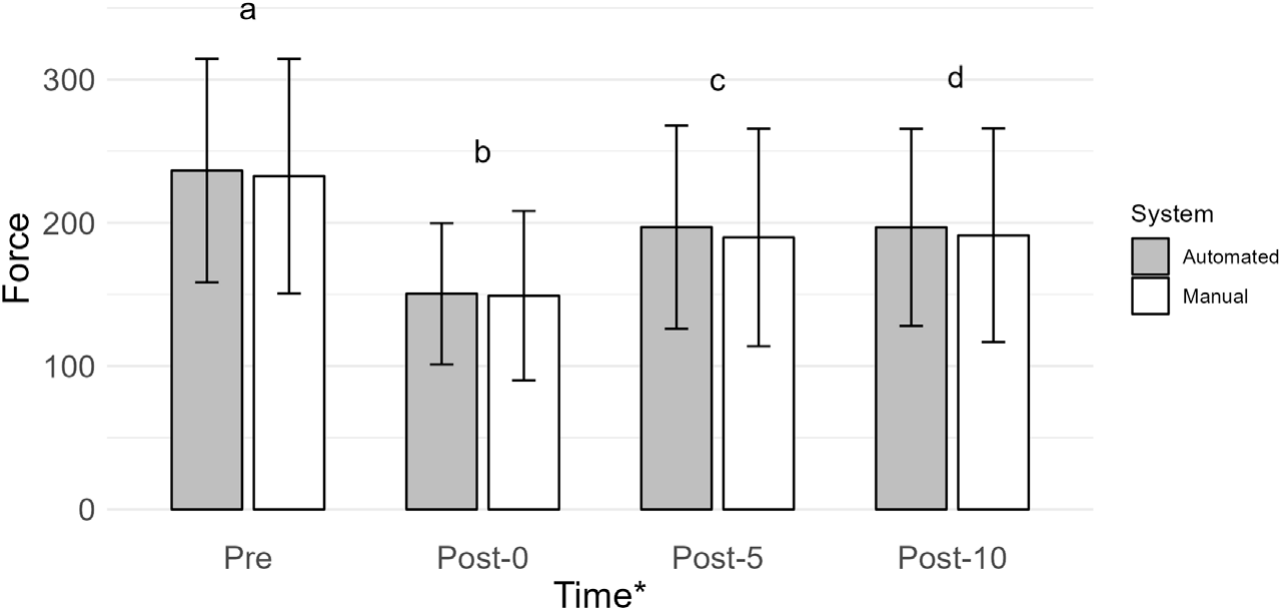
Mean force (N) is shown by vertical bars (automated, gray; manual, white), and error bars show the standard deviation. A * indicates a main effect of time. If a time point has a different lowercase letter, then there is more than moderate evidence (BF_10_ > 3), indicating a difference across time. Pre, before exercise; Post-0, immediately after exercise; Post-5, 5 min after exercise; Post-10, 10 min after exercise.

The main effect of time was the most probable model (BF_10_ = 1.246e + 29; error = 1.353%; Figure 4). Collapsed across conditions, force was greatest at Pre (234.47 ± 79.23) when compared to Post-0 (149.82 ± 52.28; BF_10_ = 2.720e + 22), Post-5 (193.34 ± 72.69; BF_10_ = 6.182e + 16), and Post-10 (194.03 ± 70.61; BF_10_ = 1.116e + 18). Force was lower at Post-0 when compared to Post-5 (BF_10_ = 1.744e + 13) and Post-10 (BF_10_ = 4.740e + 13). Force at Post-5 and Post-10 was similar (BF_10_ = 0.151). All *post hoc* comparison errors were ≤ 0.084%.

### 3.8 Repetitions (*n* = 31)

**FIGURE 5:**
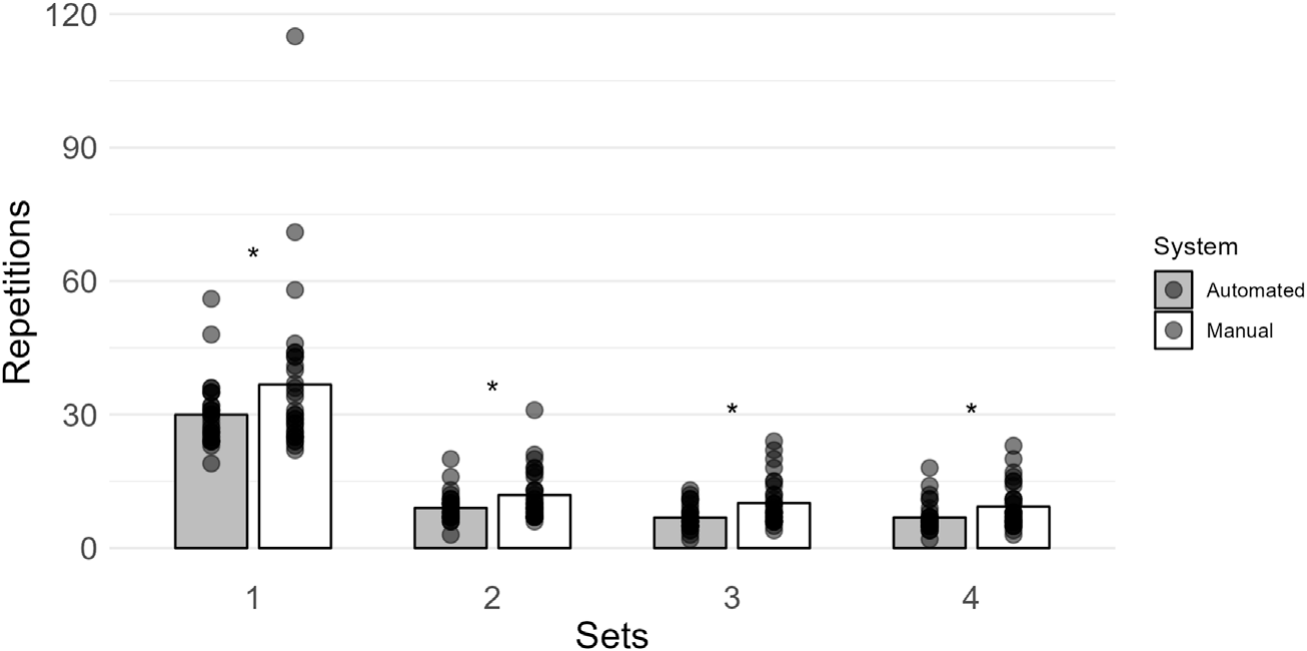
Mean repetitions within each set are shown by vertical bars (automated, gray; manual, white), and individual scores are shown with semi-transparent circles. A * indicates more than moderate evidence (BF_10_ > 3) that the manual system had more repetitions within a set.

More repetitions were completed when using the manual system (set 1: 37 ± 18; set 2: 12 ± 6; set 3: 10 ± 5; set 4: 9 ± 5) compared to the automated system (set 1: 30 ± 7; set 2: 9 ± 3; set 3: 7 ± 3; set 4: 7 ± 3) for all sets (set 1: BF_10_ = 9.698; set 2: BF_10_ = 253.112; set 3: BF_10_ = 5645.595; set 4: BF_10_ = 2122.466; Figure 5). All *post hoc* comparison errors were ≤ 8.111e − 6%.

## 4 Discussion

The current recommendation for applying BFR is to base the pressure on a percentage of AOP (Patterson et al., 2019). At rest, supine AOP was higher when using the manual system, but standing AOP prior to exercise resulted in a similar AOP between BFR systems. This was unexpected, as wider cuffs (Loenneke et al., 2013a; Jessee et al., 2016; Buckner et al., 2017) and less flexible cuff materials typically result in a lower AOP (Buckner et al., 2017). Additionally, while previous research has found that a seated position may result in a greater AOP than a supine position (Sieljacks et al., 2018), we expected the cuff components to affect AOP similarly in the supine or standing position. Future research should investigate whether body position affects the manual or automated AOP assessment differently, as presumed in this study.

The first part of our experiment investigated the resting cardiovascular response to BFR, as any differences could result in a different stimulus during exercise. Our findings suggest that either system, when applied relative to AOP at rest, causes a similar change in blood flow, oxygen saturation, and heart rate. Previous applications of BFR found that an inflated wider cuff had a higher systolic blood pressure at rest, and an inflated narrow cuff increased the heart rate (Rossow et al., 2012). However, the pressure applied to each cuff width was the same (130% of brachial systolic blood pressure) and likely resulted in a greater restriction stimulus from the wider cuff. In the current study, we applied a pressure that would allow blood flow at 50% AOP, as measured by the system used. This appears to have a similar cardiovascular response between cuffs of different widths than pressures applied in the lower body based on brachial systolic blood pressure. Current applications of relative BFR (% AOP) elicit a similar decrease in blood flow at rest, regardless of cuff width (Mouser et al., 2017). In the current study, we found that our baseline blood flow and 50% AOP blood flow were comparable to the values found in Mouser et al. (2017). Even BFR pressures using elastic wraps can be made relative to arm circumference and have reduced blood flow, similarly to pneumatic systems (Abe et al., 2019). Thus, it seems as though differences between systems, cuffs, or individuals may be accounted for with the use of a relative BFR pressure based on the BFR system used at rest.

The systems used for BFR exercise under relative pressures appear to result in similar acute muscular responses but may reach momentary failure at different times. Acute increases in muscle thickness, thought to indicate muscle swelling, may be a mechanism for muscle growth (Yasuda et al., 2012), whereas changes in muscular force can be indicative of muscular fatigue (Nocella et al., 2011), indicating the strength of the exercise stimulus. Following BFR exercise, force remained below baseline and muscle thickness remained above baseline 5 and 10 min into recovery, regardless of the system used. Our findings suggest that either system causes similar muscle swelling and fatigue following exercise taken to failure. These findings are in line with those of previous studies with BFR exercise until momentary failure. For instance, muscle swelling occurs immediately after exercise and remains into recovery (Jessee et al., 2017b; Jessee et al., 2018b; Jessee et al., 2019), and there is an immediate decline in muscular force that can remain below baseline for at least 30 min (Jessee et al., 2017b). When applying 50% AOP and exercising to momentary failure with either system, it is likely that the muscular adaptations would be similar if repeated over the course of a training regimen. When training chronically, muscle growth is similar across conditions (Jessee et al., 2018c), even when some conditions have greater acute muscle swelling responses (Buckner et al., 2019). However, momentary failure can be reached in more repetitions with the manual system than with the automated system. With this consideration, a predetermined set and repetition protocol (i.e., 30 × 15 × 15 × 15) with an automated BFR system may make achieving goal repetitions more difficult or lead to different responses.

Perceived discomfort may be an important factor in why the manual system resulted in more repetitions than the automated system. During the exercise, we found that RPE-D increased over time with both systems and was greater with the automated system. Although this is consistent with BFR research investigating different cuff widths (Loenneke et al., 2014; Spitz et al., 2019; 2021), it is in contrast with work comparing automated systems that regulate cuff pressure during exercise (Jacobs et al., 2023). BFR exercise of the upper body may be more sensitive to discomfort than the lower body. During a concentric muscle action, the muscle shortens under the BFR cuff and may occur to a greater extent in the upper body, potentially causing more discomfort. In the upper body, wider cuffs of the same material and system have greater discomfort than their narrower counterparts (Spitz et al., 2019). However, RPE-D during lower-body BFR exercise is similar between wide and narrow cuffs (Spitz et al., 2021). Taking these components into account, it seems that pressure regulation during upper-body exercise does not offset the discomfort of a system with a wider cuff. However, when exercising to momentary failure with BFR, it appears that RPE-E increases during exercise, regardless of the system used. This is consistent when comparing two manual systems (Buckner et al., 2017), but the effects of automated systems appear to differ across studies. For example, an automated system that regulates cuff pressure resulted in higher RPE-E than a non-regulated system (Bordessa et al., 2021), which is in contrast to Jacobs et al. (2023). Although the automated system may be more convenient for clinical application, it may result in greater discomfort during exercise without having an additive change in the acute muscular responses.

### 4.1 Limitations

This study is not without limitations; our AOP measurement in the standing position measured each arm with a different system and could be impacted by interarm differences. However, interarm differences in blood pressure are generally very small in a healthy population (Sharma and Ramawat, 2016). One condition per arm has been utilized in previous studies (Abe et al., 2019; Spitz et al., 2019), with AOP assumed to be similar between arms. Additionally, standing AOP measures typically occur immediately following seated rest (Jessee et al., 2016; Buckner et al., 2017), but measures of muscle thickness prior to AOP measurement in this study may have had individuals in the standing position longer than in typical studies. However, with these limitations, there is evidence that standing AOP is similar between these systems, given their numerous differences. The time to reach AOP was not assessed in this study; however, it is unlikely that this would have affected the measurement, as it was consistent across participants. These results are only acute responses and applicable to the upper body in a healthy population, and they may not be generalizable to older or clinical populations. We did not explore sex differences nor was the menstrual cycle assessed in this study.

### 4.2 Conclusion

Overall, the acute cardiovascular response to BFR is similar between manual and automated systems using the same relative AOP at rest. The exercise response is mostly similar, although the automated system may augment discomfort and limit repetitions to momentary failure in the upper body. Thus, research on the response to resting or exercise to BFR using the manual system may have similar interpretations to the automated system if the protocols and relative pressures are similar. While the manual system can be used with a narrower cuff option, the automated system requires less equipment to apply relative pressures. When determining which system to utilize for BFR, users should consider the participants, resources, and outcomes of interest.

## Data Availability

The raw data supporting the conclusion of this article will be made available by the authors, without undue reservation.
